# QTL analysis for ascorbic acid content in strawberry fruit reveals a complex genetic architecture and association with GDP-L-galactose phosphorylase

**DOI:** 10.1093/hr/uhad006

**Published:** 2023-01-19

**Authors:** Pilar Muñoz, Cristina Castillejo, José Antonio Gómez, Luis Miranda, Silke Lesemann, Klaus Olbricht, Aurélie Petit, Philippe Chartier, Annika Haugeneder, Johanna Trinkl, Luca Mazzoni, Agnieszka Masny, Edward Zurawicz, Freya Maria Rosemarie Ziegler, Björn Usadel, Wilfried Schwab, Béatrice Denoyes, Bruno Mezzetti, Sonia Osorio, José F Sánchez-Sevilla, Iraida Amaya

**Affiliations:** Centro IFAPA de Málaga, Instituto Andaluz de Investigación y Formación Agraria y Pesquera (IFAPA), 29140, Málaga, Spain; PhD program in Advanced Biotechnology, Universidad de Málaga, 29071, Málaga, Spain; Centro IFAPA de Málaga, Instituto Andaluz de Investigación y Formación Agraria y Pesquera (IFAPA), 29140, Málaga, Spain; Finca el Cebollar, Centro IFAPA las Torres, 04745, Huelva, Spain; Finca el Cebollar, Centro IFAPA las Torres, 04745, Huelva, Spain; Hansabred GmbH & Co. KG, 01108, Dresden, Germany; Hansabred GmbH & Co. KG, 01108, Dresden, Germany; INVENIO, 33800, Bordeaux, France; INVENIO, 33800, Bordeaux, France; Biotechnology of Natural Products, Technische Universität München, 85354, Freising, Germany; Biotechnology of Natural Products, Technische Universität München, 85354, Freising, Germany; Dipartimento di Scienze Agrarie, Alimentari e Ambientali, Università Politecnica delle Marche, 60131, Ancona, Italy; Department of Horticultural Crop Breeding, the National Institute of Horticultural Research, Konstytucji 3 Maja 1/3, 96-100, Skierniewice, Poland; Department of Horticultural Crop Breeding, the National Institute of Horticultural Research, Konstytucji 3 Maja 1/3, 96-100, Skierniewice, Poland; Institute of Bio- and Geosciences, Bioinformatics (IBG-4), Forschungszentrum Jülich GmbH, 52428, Jülich, Germany; Institute of Bio- and Geosciences, Bioinformatics (IBG-4), Forschungszentrum Jülich GmbH, 52428, Jülich, Germany; Biotechnology of Natural Products, Technische Universität München, 85354, Freising, Germany; Univ. Bordeaux, INRAE, Biologie du Fruit et Pathologie, UMR 1332, F-33140, France; Dipartimento di Scienze Agrarie, Alimentari e Ambientali, Università Politecnica delle Marche, 60131, Ancona, Italy; Departamento de Biología Molecular y Bioquímica, Instituto de Hortofruticultura Subtropical y Mediterránea "La Mayora", Universidad de Málaga-Consejo Superior de Investigaciones Científicas, Campus de Teatinos, 29071 Málaga, Spain; Unidad Asociada de I+D+i IFAPA-CSIC Biotecnología y Mejora en Fresa, 29010, Málaga, Spain; Centro IFAPA de Málaga, Instituto Andaluz de Investigación y Formación Agraria y Pesquera (IFAPA), 29140, Málaga, Spain; Unidad Asociada de I+D+i IFAPA-CSIC Biotecnología y Mejora en Fresa, 29010, Málaga, Spain; Centro IFAPA de Málaga, Instituto Andaluz de Investigación y Formación Agraria y Pesquera (IFAPA), 29140, Málaga, Spain; Unidad Asociada de I+D+i IFAPA-CSIC Biotecnología y Mejora en Fresa, 29010, Málaga, Spain

## Abstract

Strawberry (*Fragaria* × *ananassa*) fruits are an excellent source of *L*-ascorbic acid (AsA), a powerful antioxidant for plants and humans. Identifying the genetic components underlying AsA accumulation is crucial for enhancing strawberry nutritional quality. Here, we unravel the genetic architecture of AsA accumulation using an F_1_ population derived from parental lines ‘Candonga’ and ‘Senga Sengana’, adapted to distinct Southern and Northern European areas. To account for environmental effects, the F_1_ and parental lines were grown and phenotyped in five locations across Europe (France, Germany, Italy, Poland and Spain). Fruit AsA content displayed normal distribution typical of quantitative traits and ranged five-fold, with significant differences among genotypes and environments. AsA content in each country and the average in all of them was used in combination with 6,974 markers for quantitative trait locus (QTL) analysis. Environmentally stable QTLs for AsA content were detected in linkage group (LG) 3A, LG 5A, LG 5B, LG 6B and LG 7C. Candidate genes were identified within stable QTL intervals and expression analysis in lines with contrasting AsA content suggested that *GDP-L-Galactose Phosphorylase FaGGP(3A),* and the chloroplast-located AsA transporter gene *FaPHT4;4(7C)* might be the underlying genetic factors for QTLs on LG 3A and 7C, respectively. We show that recessive alleles of *FaGGP(3A)* inherited from both parental lines increase fruit AsA content. Furthermore, expression of *FaGGP(3A)* was two-fold higher in lines with high AsA. Markers here identified represent a useful resource for efficient selection of new strawberry cultivars with increased AsA content.

## Introduction

Vitamin C, or ascorbic acid (AsA), is one of the most abundant water-soluble antioxidants synthesized in all major groups of organisms, including plants and animals. AsA plays important biological roles as an enzyme cofactor, a radical scavenger, and a donor/acceptor in electron transport either at the plasma membrane or in the chloroplasts (for review, see [1–3]). In plants, it is involved in different processes, including plant development, cell division and expansion, hormone signaling, and scavenging reactive oxygen species, and thus protecting DNA, proteins or lipids from oxidative damage during photosynthesis or abiotic stresses [1, 3].

Due to mutations in the gene encoding the enzyme *L*-gulono-1,4-lactone oxidase, which catalyzes the last step of the animal biosynthetic pathway, humans and some animals are unable to synthesize AsA [4], becoming an essential vitamin in our diet. AsA plays an important role in human health, as it is involved in the synthesis of collagen and therefore in the maintenance of cartilage and bones, of a healthy immune system and in the prevention of cardiovascular diseases [1, 5]. The primary source of vitamin C in our diet comes from plant-synthesized ascorbic acid, mainly through fresh fruit and vegetable consumption. However, ascorbic acid content varies among different species, with high amounts found in kiwifruit, strawberry, citrus fruits and some vegetables such as broccoli [1, 6].

**Figure 1:**
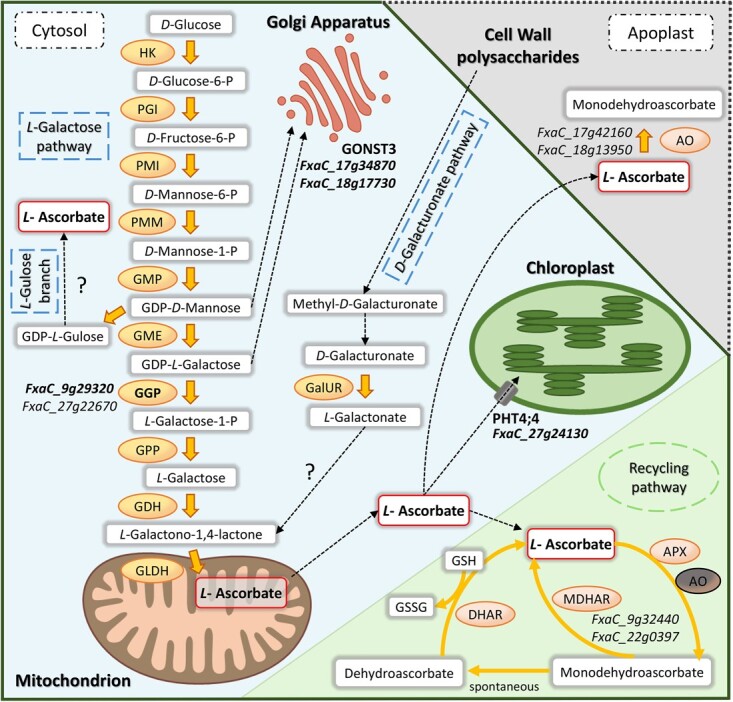
Major biosynthesis and recycling pathways for ascorbic acid (AsA) content in plants and candidate genes for AsA content in strawberry fruit detected in the QTL intervals based on *Fragaria × ananassa* physical position. HK, Hexokinase; PGI, phosphoglucose isomerase; PMI, phosphomannose isomerase; PMM, phosphomannosemutase; GMP, GDP-*D*-mannose pyrophosphorylase; GME, GDP-*D*-mannose 3′, 5′ epimerase; GGP, GDP-*L*-galactose-phosphorylase, GPP, *L*-galactose-1-phosphate phosphatase; GDH, *L*-galactose dehydrogenase; GLDH, *L*-galactono-1,4-lactone dehydrogenase; AO, ascorbate oxidase; APX, ascorbate peroxidase; MDHAR, monodehydroascorbate reductase; DHAR, dehydroascorbate reductase; GalUR, *D*-galacturonate reductase; GONST3, Golgi localized Nucleotide Sugar Transporter (GDP-*L*-galactose transporter); PHT4;4, Ascorbate transporter; GSH, glutathione; GSSG, oxidized glutathione.

AsA concentration is determined by the balance between biosynthesis, oxidation, enzymatic regeneration or recycling, and transport. Different biosynthetic pathways have been described in plants, although the Smirnoff-Wheeler or *L*-galactose pathway is considered the dominant route in many species [7, 8]. This pathway takes place in the cytosol except for the last step, which occurs in the intermembrane space of the mitochondrion. All the genes involved in this pathway, which starts from *D*-glucose and has *D*-mannose and *L*-galactose as intermediates, have been characterized (Figure 1). Different studies in *Arabidopsis* have shown that *GDP-L-galactose-phosphorylase* (*GGP*) is a crucial point of regulation in the pathway, being tightly controlled at the transcriptional and translational levels [9–12]. Several studies have also reported the importance of GGP in regulating AsA content in different tissues (vegetative, tubers or fruits) of diverse species, such as lettuce [13], apple [14], tomato, potato or strawberry [15–17].

Alternative AsA biosynthetic pathways have been described in plants, but their relevance seems to depend on the species and the developmental stage of the plant or tissue [3, 18]. The enzyme GDP-*D*-mannose 3′,5′-epimerase (GME) from the Smirnoff-Wheeler pathway also catalyzes the 5′-epimerization of GDP-*D*-mannose to produce GDP-*L*-gulose, which could initiate an alternative branch, the *L*-gulose pathway [19]. Although still under question, this and other evidence support the existence but not the relevance of this animal-like pathway in plants. The best characterized alternative route in plants is the *D*-galacturonate pathway, which uses pectin from cell wall degradation via the enzyme *D*-galacturonate reductase (GalUR) to complete AsA production in fruits such as tomato [20], strawberry [21], or apple [14]. Another alternative AsA biosynthetic pathway is through oxidation of myo-inositol to *D*-glucuronate by a myo-inositol oxidase [22]. However, there is much controversy about whether this route significantly affects AsA accumulation as it might be predominantly involved in hexose, starch and pectin metabolism [23, 24].

As a strong antioxidant, AsA can accept electrons from a wide range of free radicals and by the action of enzymes as ascorbate peroxidase (APX) or ascorbate oxidase (AO). To preserve the redox cellular homeostasis, ascorbate oxidized forms, monodehydroascorbate (MDHA) and dehydroascorbate (DHA), undergo enzymatic regeneration by enzymes monodehydroascorbate reductase (MDHAR) and dehydroascorbate reductase (DHAR) through the ascorbate-glutathione cycle (Figure 1) [25]. Transport of AsA and its intermediates are also crucial in AsA metabolism. AsA transporters found in cell membranes maintain AsA concentrations in different organelles and cellular spaces [26, 27].

Strawberry (*Fragaria* × *ananassa* Duch.) is an allo-octoploid species (2n = 8× = 56) originating from the hybridization in a European garden of two octoploid wild species; the south American *Fragaria chiloensis* and the north American *F. virginiana* [28, 29]. The octoploid strawberry genome has recently been sequenced [30]. The genome of these octoploid species is formed by four subgenomes derived from four diploid progenitor species. Two of the four subgenomes come from *F. vesca* and *F. iinumae*, whereas the origin of the other two is still under investigation [30–33]. Strawberry fruit is highly consumed and appreciated for its flavor, aroma and nutritional value [34]. In general, strawberry is rich in AsA, although its content varies among species of the *Fragaria* genus and also between strawberry cultivars [35–38]. Over the last decades, strawberry breeding has focused on maintaining high yield and increasing resistance to pests while improving fruit firmness and flavor [39–41]. Focusing on these important targets could indirectly have a negative impact on fruit AsA content in new breeding selections. Due to increasing consumer demand, enhancing fruit organoleptic and nutritional quality are becoming current breeding targets [36, 39, 41]. Genetic characterization of fruit AsA regulation would allow the identification of markers and genes potentially useful for trait improvement, not only for enhancing the nutritional value of strawberry but also for stimulating stress tolerance.

The genetic control of AsA content in fruits has been well studied in tomato [42, 43] and apple [14, 44] and several candidate genes were identified through quantitative trait locus (QTL) mapping and functional studies. Reports in both species have shown that AsA content in fruit exhibits a quantitative inheritance with many loci involved in its regulation. Previous genetic studies in strawberry have used the 232 × 1392 biparental population derived from two breeding lines with similar Mediterranean pedigree [38, 45]. Only three QTLs explaining in total a large (45%) variation were detected on chromosomes 4C (LG IV-2), 5C (LG V-1) and 7A (LG VII-1). In this study, we have characterized the Can×SS F_1_ population derived from parental lines ‘Candonga’ (Can) and ‘Senga Sengana' (SS), representing European breeding history. Cultivar ‘Candonga’ was developed in 2003 and it is well adapted to Mediterranean areas, producing high yields and firm fruit, while ‘Senga Sengana' is an older German cultivar selected around 1944 and introduced on the market in 1952. “Senga Sengana” is well adapted to middle, eastern and northern Europe, as well as to the east coast of North America, and produces smaller and softer fruit with good flavor. This segregating population was first used to evaluate the variance of AsA content in strawberry fruit, as well as its interaction with the environment and crop management. Secondly, it was used to identify QTLs in five different environments and candidate genes affecting AsA content in cultivated strawberry fruit. Lastly, we have found an association between allelic variants of *GDP-L-galactose phosphorylase* and its expression levels in lines with contrasting AsA content. Kompetitive Allele Specific PCR (KASP) markers developed in this study represent a useful tool to assist in the selection of new strawberry cultivars with high AsA content.

## Results

### Environmental and genetic variation of ascorbic acid content in strawberry fruit

**Figure 2:**
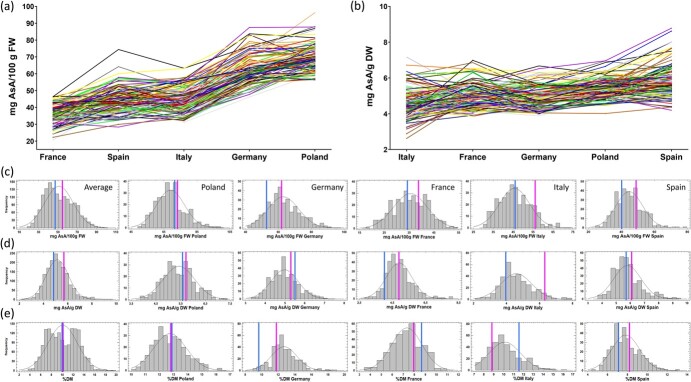
Fruit ascorbic acid (AsA) content in mg AsA/100 g FW (a) or mg AsA/g DW (b) in the Can×SS population grown in the five countries. Each color line represents AsA content from an independent F_1_ line and parentals. Countries are ordered from the lowest mean AsA content to the highest. Distribution of AsA content in the Can×SS population in mg AsA/100 g FW (c), in mg AsA/g DW (d), and content of Dry Matter (%DM; e). Mean parental values are shown in magenta for ‘Candonga’ and in blue for ‘Senga Sengana’.

Preliminary analysis during the 2016–2017 season of a subset of 35 lines of the Can×SS population, including the parental lines, revealed a great variance in AsA content (Supplementary Table S1), ranging from 35 to 90 mg per 100 g of fresh weight (FW) or 2 to 8 mg per g of dry weight (DW), and thus indicated the usefulness of this population for analysis of AsA content variation and that QTL mapping would be feasible. To evaluate the variance of AsA in the full segregating population, AsA content was measured in the parental and in the selected 113 F_1_ lines grown in five different countries during the 2017–2018 season: Poland, Germany, France, Italy and Spain (Table 1). Variation in AsA content showed significant genetic and environmental effects in all countries (Supplementary Table S2; Figure 2). As shown in Table 1 and Figure 2, AsA content ranged from 18 to 97 mg/100 g FW or 2 to 10 mg/g DW. Mean AsA content varied significantly among countries indicating a clear environmental effect (Table 1). Significant interaction between environment and genotype was also detected by ANOVA (Supplementary Table S2), although, as depicted in Figure 2a and b, genotypes with higher or lower AsA content were in general consistent in the five countries. In agreement, and as detailed later on, significant positive correlations were found in AsA content among all countries (Supplementary Fig. S1). AsA content expressed in fresh or dry weight and dry matter (DM) content followed a normal distribution in most countries (Figure 2c, 2d and 2e), suggesting a complex quantitative nature for these traits. Dry matter content also varied among individuals (Figure 2e), showing genotypic and environmental effects and genotype × country interaction (Supplementary Table S2).

**Table 1:** Variation of fruit ascorbic acid (AsA) content in the Can×SS population in the 2017–2018 season. Least square mean (± standard deviation (sd)), number of data (n), p-value (p) of differences between ‘Candonga’ and ‘Senga Sengana’ and range of AsA content in mg AsA/100 g FW, mg AsA/g DW and % dry matter (DM) in the five countries. Different superscripts indicate significant differences (p-value <0.05; tested by ANOVA and Student–Newman–Keuls)

	**Country**	Germany	Spain	France	Italy	Poland
	**n**	341	343	333	337	287
	**Candonga**	62.35	49.27	38.86	56.06	73.52
**AsA** **(mg/100 g FW)**	**Senga Sengana**	53.02	40.05	34.73	46.00	72.00
	**p**	*	*	ns	*	ns
	**Population Mean (± sd)**	62.89 ± 9.40^d^	44.76 ± 8.08^b^	35.52 ± 6.86^a^	45.65 ± 7.61^c^	68.98 ± 8.43^e^
	**Population Range**	42.04–91.10	25.53–77.49	18.28–53.49	30.66–67.60	51.31–97.28
	**Candonga**	5.35	6.08	4.88	6.36	5.73
	**Senga Sengana**	5.52	5.70	4.02	3.99	5.57
**AsA** **(mg/g DW)**	**p**	ns	ns	*	*	ns
	**Population Mean (± sd)**	4.98 ± 0.69^b^	5.79 ± 0.97^d^	4.90 ± 0.77^b^	4.59 ± 0.93^a^	5.43 ± 0.64^c^
	**Population Range**	3.35–7.04	3.76–9.54	2.94–8.03	2.39–7.91	3.89–7.47
	**Candonga**	11.67	8.20	7.96	8.81	12.85
	**Senga Sengana**	9.61	7.14	8.63	11.59	12.92
**%DM**	**p**	*	ns	ns	*	ns
	**Population Mean (± sd)**	12.70 ± 1.55^d^	7.82 ± 1.38^b^	7.32 ± 1.38^a^	10.11 ± 1.60^c^	12.75 ± 1.16^d^
	**Population Range**	8.99–18.91	4.48–13.12	3.94–11.62	7.15–16.13	10.10–16.89

Depending on how data was expressed, relative to FW or DW, mean AsA values varied differently between countries (Table 1; Figure 2a and 2b). Poland and Germany showed the highest AsA content relative to FW, while the content was lowest in Spain and France (countries with lowest values of DM). However, when AsA was expressed as mg/g DW, Spain and Poland had the highest AsA content, followed by France and Germany, while Italy displayed lower values. In general, AsA content was higher in ‘Candonga’ than in ‘Senga Sengana’, with the only exception found for AsA in mg/g DW in Germany (Table 1; Figure 2c  and 2d).

Significant positive correlations among the five countries were observed for AsA content in FW, AsA in DW and DM content (Supplementary Fig. S1). Similarly, positive correlations were observed between AsA content expressed in FW and DW in each country. On the other hand, positive correlations were observed between AsA content expressed in FW and the DM content for each country, while negative correlations were observed between AsA content expressed in DW and the DM content, suggesting that AsA content is negatively influenced by fruit water content. High broad-sense heritability (H^2^) values for the three traits were obtained for all five countries (Supplementary Table S3), indicating a large genetic effect that might facilitate QTL detection.

### Can×SS linkage map and QTL mapping

The Can×SS linkage map was constructed using SNP markers genotyped in the parental and 126 F_1_ lines using both the strawberry DArTseq platform [46] and the 50 K Fana Axiom array [47]. After filtering for high-quality polymorphic SNPs, a total of 22 960 SNP markers were initially uploaded for mapping using the *JoinMap^®^5* software [48]. Evaluation of SNP segregation ratios led to the exclusion of 11 F_1_ lines identified as selfings of ‘Candonga’ (H018, H025, H026, H034, H038, H041, H044, H048, H061, H082, and H092) and two additional F_1_ lines with high missing data and a low call rate (H069 and H111), diminishing population size to 113. Selection of non-redundant markers decreased the number to 7114 and a final elimination of markers that produced conflicting mapping resulted in a Can×SS consensus linkage map with a total of 6974 markers (Supplementary Fig. S2a; Supplementary Table S4), distributed in 6044 unique positions, of which 1879 were heterozygous markers (1:2:1). A total of 30 linkage groups (LGs) were generated in the integrated map (Supplementary Fig. S2b), two more than the expected 28 chromosomes of the cultivated strawberry. Two independent female and male parental groups (LG 2D-F and LG 2D-M) were obtained for chromosome 2D, and chromosome 4C was fragmented in a large LG 4C-I and a small LG 4C-II with only 11 markers. Marker positions in the LGs obtained for Can×SS were compared with the physical position in the *F.* × *ananassa* cv. ‘Camarosa’ v1.0 genome [30]; Supplementary Fig. S3). In general, we observed a high degree of collinearity, with many of the exceptions being reported scaffolding errors in the ‘Camarosa’ v1.0 genome assembly [49], as the major inversion on LG 2C or other detected discrepancies on 1A and 1B, 2D, 4A or 6C and D.

**Figure 3:**
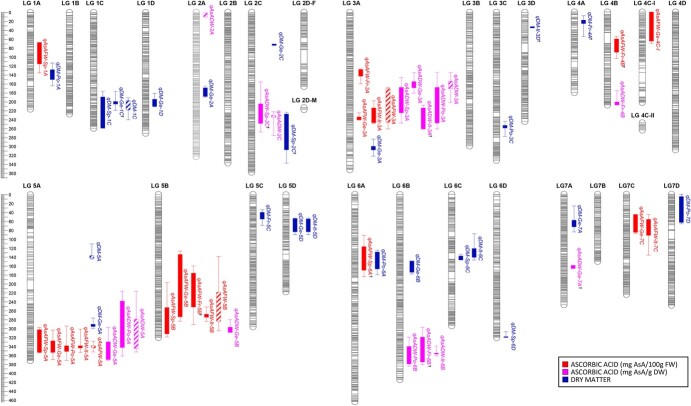
Position of QTLs controlling ascorbic acid content (in mg/100 g FW in red, and in mg/g DW in pink) and dry matter content (in blue) in strawberry fruit detected in each country (colored bars) and using the average content of the five countries (bars with stripes). *Fr,* France; *Ge,* Germany; *It,* Italy; *Po,* Poland; *Sp,* Spain. Thick and thin bars mark 1-LOD and 2-LOD QTL intervals, respectively. ^†^QTL LOD just below the threshold.

For the three evaluated traits, AsA content in FW or in DW and DM content, a total of 61 significant associations were found using restricted multiple QTL mapping (rMQM; Supplementary Table S5; Figure 3). Most significant associations were also detected by Kruskal–Wallis (Supplementary Table S5). Among them, 52 QTLs were detected using single-country data and nine using the mean values from all five countries. For AsA expressed as mg/100 g FW, 17 associations were found in the different countries, which, considering the overlapping regions as individual loci, could be summarized in eight different QTLs. Four of those QTLs (50%), on LGs 3A, 5A, 5B and 7C, were stable in at least two countries. Moreover, in three of those regions (LG 3A, 5A and 5B), QTLs were also detected using the AsA in FW average values across the five countries. The variance explained by QTLs for AsA in FW ranged from 7.5 to 24.7% (Supplementary Table S5), indicating a moderately high number of loci contributing to small portions of the observed variation. Among the four stable QTLs, *qAsAFW-5B* and *qAsAFW-7C* explained a limited percentage of variance (7.5–11.6% and 9.9–11%, respectively), while *qAsAFW-3A* and *qAsAFW-5A* explained a larger variance (16.2–24.7 and 10.9–21.7%, respectively, depending on the environment). A total of 13 associations were detected for AsA expressed as mg/g DW that could be summarized in seven different QTLs, with three of them (~ 43%) being detected in at least two countries and considered stable. The mean variance explained by AsA/DW QTLs was very similar to that for AsA/FW (Supplementary Table S5), supporting a moderately complex genetic architecture for AsA content in strawberry fruit.

Interestingly, QTLs for AsA in FW and DW from individual countries colocalized in three chromosomal regions on LGs 3A, 5A and 5B, where QTLs were also detected using the mean values of the five countries (Figure 3). In addition to these three common QTLs, a stable QTL for AsA in DW (detected in Poland, France and Italy) and a stable QTL for AsA in FW (detected in Germany and Italy) were detected on LGs 6B and 7C, respectively. Collectively, these five stable QTLs controlled an important amount of total variance: about 70% when mean values were added up.

For dry matter content, 22 different QTLs were detected, from which only three were identified in two countries (Figure 3; Supplementary Table S5), indicating a larger environmental effect on DM content in strawberry fruit. In agreement, although very similar to those for AsA content, broad-sense H^2^ were slightly lower for this trait (Supplementary Table S3). In general, we did not observe colocalization between QTLs for AsA (FW or DW) and DM, indicating that detected QTLs for AsA are not related to a variation in dry matter content.

### Effect of pyramiding AsA QTLs

**Figure 4:**
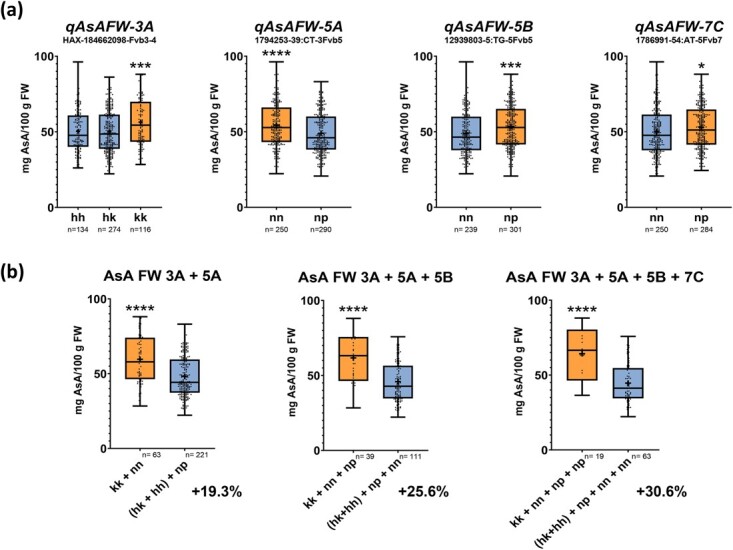
(a) Single marker analysis showing the effect on ascorbic acid (AsA) content (in mg/100 g FW) of positive (orange) and negative (blue) alleles for each stable QTL using mean phenotypic data of the five countries. (b) Effect of pyramiding positives alleles (orange) on AsA content in FW. Boxes span the 25th–75th percentiles, the middle line represents the median, and whiskers show minimum and maximum values. Dots represent individual values. Asterisks represent significant differences in mean AsA content in the five countries by ANOVA and multiple comparation (Tukey) analysis for the three possible genotypes (hh, hk, kk) of the selected marker or T-student test for the two possible genotypes (nn and np) of the selected marker (^*^: p-value <0.05; ^***^: p-value <0.001; ^****^: p-value<0.0001). *n,* number of samples.

**Figure 5:**
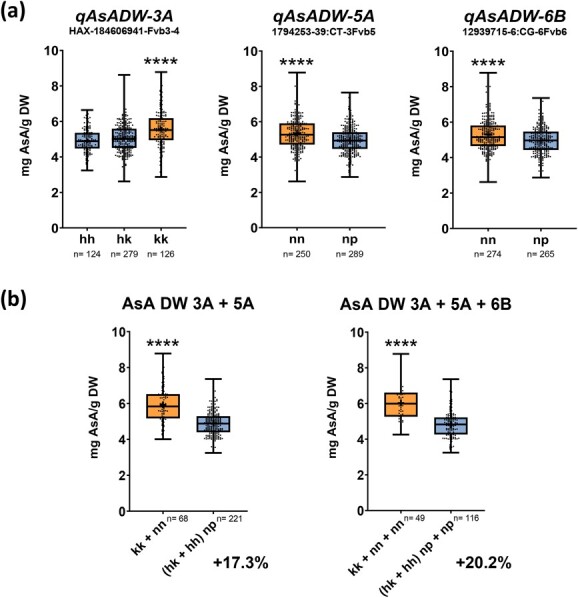
(a) Single marker analysis showing the effect on ascorbic acid (AsA) content (in mg/g DW) of positive (orange) and negative (blue) alleles for each stable QTL using mean phenotypic data of the five countries. (b) Effect of pyramiding positives alleles (orange) on AsA content in DW. Boxes span the 25th–75th percentiles, the middle line indicates the median, and whiskers show minimum and maximum values. Dots represent individual values. Asterisks represent significant differences in mean AsA content in the five countries by ANOVA and multiple comparation (Tukey) analysis for the three possible genotypes (hh, hk, kk) of the selected marker or T-student test for the two possible genotypes (nn and np) of the selected marker (^*^, p-value <0.05; ^***^, p-value <0.001; ^****^, p-value<0.0001). *n,* number of samples.

One significant SNP from each of the four stable QTLs for AsA/FW detected on LGs 3A, 5A, 5B, and 7C were assessed in a single marker analysis for association with AsA content using the average concentration from the five countries (Figure 4a). One SNP from each of the three stable QTLs for AsA/DW detected on LGs 3A, 5A and 6B were also evaluated in a single marker analysis (Figure 5a). As modeled by the MapQTL6 software (Supplementary Table S5), the QTL for AsA on LG 3A segregates as a recessive locus for high AsA content both in FW and DW. Selected markers for *qAsAFW-3A* and *qAsADW-3A* QTLs segregated 1:2:1 in the population and as expected, only the recessive homozygous genotype (kk) had a significant 10.9% (AsA/FW) and 10.5% (AsA/DW) increase in AsA content. In contrast, the alternative homozygous and heterozygous F_1_ lines had fruits with lower AsA content in FW and DW. For the rest of stable QTLs, positive alleles were found to be provided by ‘Senga Sengana’, in coupling for markers on *qAsAFW-5B* and *qAsAFW-7C*, and in repulsion for markers on *qAsAFW-5A*, *qAsADW-5A* y *qAsADW-6B* (Supplementary Table S5). Single marker effects from these QTLs ranged from a 5.6 to 9.9% increase in AsA content. Although the effect of every single marker on AsA content is modest, pyramiding positive alleles increased AsA/FW by 19.3% with the two markers with the highest effects, 25.6% with the third one and by 30.6% combining the four stable QTLs for AsA in FW (Figure 4b). For QTLs affecting AsA in mg/g DW, the two selected markers from larger effect QTLs, *qAsADW-3A* and *qAsADW-5A*, increased AsA content by 17.3%, and adding up *qAsADW-6B* QTL, further elevated AsA content to 20.2% (Figure 5).

### Identification of candidate genes in QTL intervals

To detect candidate genes underlying the five AsA QTLs that were stable in different environments, we searched annotations within 1-LOD intervals using the *F.* × *ananassa* ‘Camarosa’ v1.0 genome [30]. Genome annotations were searched for known genes related to AsA biosynthesis, recycling or transport and reported transcription factors, such as *MYBS1* and *GBF,* regulating its concentration [3, 18, 50, 51]. 1-LOD intervals were in general large and enclosed a total number of transcripts ranging from 576 for *qAsADW-6B* to 1283 in the overlapping interval for *qAsAFW-3A* and *qAsADW-3A* (Supplementary Table S6). Two candidate genes were detected in the QTL interval on LG 3A (*qAsAFW-3A* and *qAsADW-3A*) which controlled a mean 19% variance (Supplementary Table S5). One of these candidate genes showed homology to a *monodehydroascorbate reductase* (*MDHAR;* maker-Fvb3–4-snap-gene-181.50) and the other to a *GDP-L-galactose phosphorylase* (*GGP;* maker-Fvb3–4-augustus-gene-163.49). On LGs 5A and 5B, stable QTLs for both AsA/FW and AsA/DW were detected in homoeologous regions and can therefore be considered homoeo-QTLs (Figure 3) as previously described for other QTLs in strawberry [38, 52]. In agreement, two candidate homoeologous genes were detected in each chromosome: a *Go**lgi-localizedNDP-Sugar Transporter* (*GONST*) with high similarity to the *GDP-galactose transporter GONST3/GGLT1* (maker-Fvb5–1-augustus-gene-177.22 on LG 5A and maker-Fvb5–3-augustus-gene-116.30 on LG 5B) and a *L-ascorbate oxidase* (*AO*; maker-Fvb5–1-augustus-gene-217.42 on LG 5A and maker-Fvb5–3-snap-gene-89.30 on LG 5B). A seventh candidate gene with homology to a *MDHAR* (maker-Fvb6–3-augustus-gene-20.23) was identified in the interval for the stable QTL *qAsADW-6B* detected in Poland, France and Italy. Finally, two additional candidate genes showing homology to another *GGP* (augustus_masker-Fvb7–1-processed-gene-166.6) and to the ascorbate transporter *PHT4;4* (maker-Fvb7–1-snap-gene-175.49), were identified in the *qAsAFW-7C* QTL detected in Germany and Italy.

We next examined whether candidate genes were expressed in ripe strawberry fruit using RNAseq data previously reported [53] but here mapped to the ‘Camarosa’ reference genome [30]. Among the nine candidate genes, only five, *FaGGP(3A)*, *FaGONST3(5A)*, *FaGONST3(5B)*, *FaMDHAR(6B)* and *FaPHT4*;*4(7C)*, one in each QTL, were significantly expressed in ‘Camarosa’ ripe fruit (Supplementary Fig. S4). The expression levels during ‘Camarosa’ fruit ripening were below 5 FPKM for all of them, except for *FaGGP(3A)*, which was predominantly expressed in white and turning receptacles. As previously described for *GGP* genes in strawberry [35, 54, 55] and other species [3, 12], high *FaGGP(3A)* expression was also detected in leaf tissue (Supplementary Fig. S4). ‘Camarosa’ subgenomes C and D harbor two additional *FaGGP* homoeologs both displaying similar expression patterns to *FaGGP(3A).*

### Expression analysis of candidate genes in contrasting F_1_ lines

**Figure 6:**
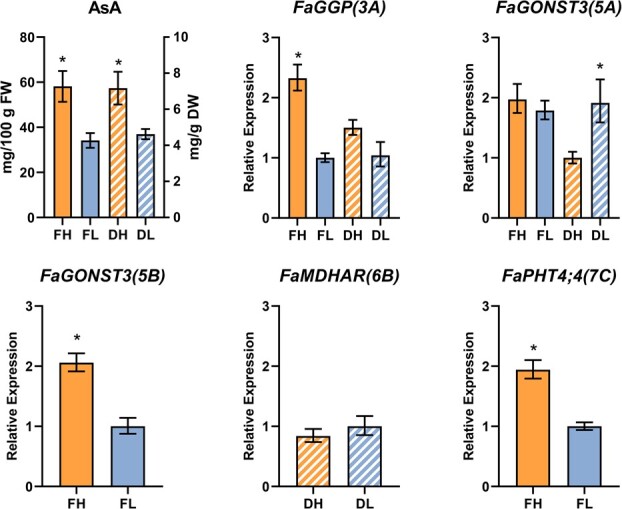
Relative expression by qRT-PCR of candidate genes in pools of fruit contrasting in ascorbic acid (AsA) content relative to FW (solid bars) or DW (dashed bars). AsA content in the different pools is shown in the first graph. FH, High AsA/FW pool; FL, Low AsA/FW pool; DH, High AsA/DW pool; DL, Low AsA/DW pool. *indicates significant differences (p-value<0.05; tested by ANOVA simple and Tukey).

To gain further insight into the putative role of *FaGGP(3A)*, *FaGONST3(5A)*, *FaGONST3(5B)*, *FaMDHAR(6B)* and *FaPHT4;4(7C)* on the regulation of AsA content, we quantified their expression levels by qRT-PCR in ripe fruit of two contrasting pools of F_1_ lines, one contrasting for AsA/FW and another for AsA/DW. High-AsA pools presented a significant 1.7 or 1.6-fold increase in AsA content per FW or DW, respectively (Figure 6). As strawberry is an allo-octoploid we considered the presence of other homoeologous copies to design subgenome-specific primers (Supplementary Table S7). The expression of *FaGGP(3A)* showed a 2-fold increase in fruits with higher AsA content in FW (Figure 6). Although the same trend was observed in the pools contrasting in AsA in DW, the difference was not statistically significant. Together with the location of *FaGGP(3A)* within the QTL on LG 3A, the positive correlation between *FaGGP(3A)* expression level and AsA content points to a significant role of *FaGGP(3A)* in determining AsA content in strawberry fruit.

Intriguingly, the two homoeologous genes on LG 5A and 5B [*FaGONST3(5A)* and *FaGONST3(5B)*] showed contrasting expression patterns (Figure 6). While *FaGONST3(5A)*, located in the overlapping QTL interval of *qAsAFW-5A* and *qAsADW-5A,* was upregulated in fruits with lower AsA/DW, the homoeologous copy on LG 5B, *FaGONST3(5B)*, was induced by 2-fold in fruits of F_1_ lines with higher AsA/ FW. *FaMDHAR(6B)* expression in ‘Camarosa’ tissues was very low (Supplementary Fig. S4) and showed no significant differences between ripe fruits of contrasting lines in the Can×SS population (Figure 6), suggesting that it might not be the gene underlying *qAsADW-6B*. Interestingly, transcript levels of the candidate gene *FaPHT4;4(7C)*, showing high protein sequence similarity to an ascorbate transporter [26], were significantly higher in fruits with higher AsA content (Figure 6), suggesting a role for this gene in the regulation of fruit AsA content in strawberry.

### Effect of different haplotypes of *FaGGP(3A)* on AsA content

**Figure 7:**
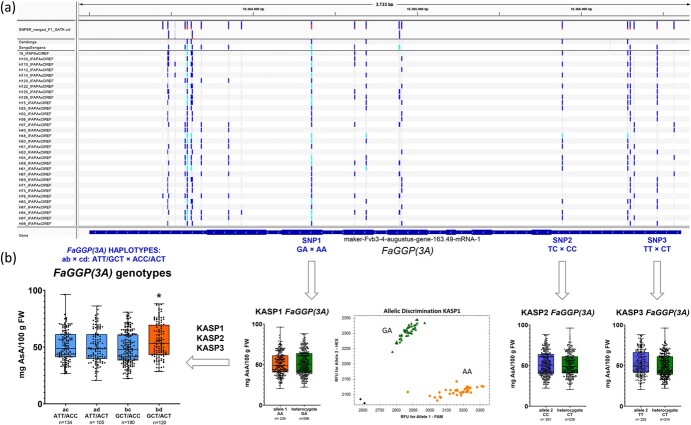
Association between fruit ascorbic acid (AsA) content and *FaGGP(3A)* allelic variants in the Can×SS population. (a) Polymorphisms in *FaGGP(3A)* transcript sequence (in the minus strand of ‘Camarosa’ genome) viewed in the Integrative Genomics Viewer (IGV). The three SNPs used for marker development are highlighted in blue. Each bar across the top of the plot shows the allele fraction for each SNP (Dark blue portion, reference allele; Red portion, alternative allele). The genotypes for each SNP in each sample are labelled grey, dark blue and light blue for reference, heterozygous or alternative SNP alleles; Lines H025, H048 and H061 are selfings of ‘Candonga’. (b) Effects on AsA content associated with each SNP and with the four haplotype combinations segregating in the population. A scatter plot for KASP1 marker is shown with genotypes colored green for heterozygous and yellow for homozygous for the FAM-A allele; black dots represent non-template controls. Boxes are delimited by 25th and 75th quartiles. Dots show individual values and whiskers represent highest/lowest values; horizontal lines represent medians. Statistical significance was determined using Student’s t test for each KASP and by ANOVA and Tukey’s test for *FaGGP(3A)* diplotypes (^∗^p-value <0.05).

Based on the differential expression of *FaGGP(3A)* in lines with low and high AsA content (Figure 6) and its importance as the control point in the main AsA biosynthesis pathway (Figure 1) [9, 11], we have further investigated the involvement of this particular homoeolog in the control of strawberry AsA content. To study the association of particular alleles of *FaGGP(3A)* with AsA content in the population, we used RNAseq data obtained from ripe fruits of ‘Candonga’, ‘Senga Sengana’ and a subset of 32 F_1_ lines (unpublished data) to search for SNPs/indels within the transcript annotation. A total 24 polymorphisms were identified in *FaGGP(3A)* mRNA and all of them were heterozygous in only one of the parental lines, segregating as a back-cross in the 32 F_1_ lines (Supplementary Table S8; Figure 7a). Three high quality SNPs were selected to test their association with AsA content, for which subgenome-specific Kompetitive Allele-Specific PCR (KASP) markers (SNP1–3) were designed and used to genotype the whole Can×SS population (Figure 7b). SNP1 and SNP2 were inherited from ‘Candonga’ while SNP3 was inherited from ‘Senga Sengana’. As expected for a recessive QTL (Supplementary Table S5) with a segregation type ab×cd, none of the three selected SNPs in *FaGGP(3A)* was associated with AsA content (Figure 7b).

However, taken together, the three SNPs defined four haplotypes for *FaGGP(3A)* (named a-d) segregating in the Can×SS population. Interestingly, the mean AsA content of each of the four genotypes (ac, 51 mg/100 g FW; ad, 50.9 mg/100 g FW; bc, 49.4 mg/100 g FW and bd, 55.8 mg/100 g FW) was extremely similar to the mean effects estimated by the MapQTL6 software for significant markers (Supplementary Table S5) and, as predicted for the QTL, only the recessive bd genotype (GCT/ACT) of *FaGGP(3A)* showed significantly higher (9.7%) AsA content in fruit (Figure 7b).

### Marker AX-184662098 in *qAsAFW-3A* is useful for marker-assisted selection

To test the usefulness of KASP markers on *FaGGP(3A)* for predicting AsA content, we genotyped 126 diverse accessions of cultivated strawberry that included cultivars from different breeding programs across the world, from different periods and some hybrids with *F. chiloensis* (Supplementary Table S9). The three SNPs on *FaGGP(3A)* defined six haplotypes that were combined into 13 different diplotypes. The number of accessions in the different genotypic classes ranged from 1 to 25. Only 7 accessions had the bd genotype, associated with higher AsA in the Can×SS population, and no significant differences in AsA content were observed between any of the genotypes. This result indicates that KASP markers in *FaGGP(3A)* are not predictive in wider germplasm, although it is possible that the low number of accessions for each genotype is limiting statistical power.

We also tested the capacity of the previously selected SNP on the stable QTL *qAsAFW-3A* (AX-184662098) to predict high AsA content in the diverse accessions. For that, we genotyped the 126 accessions with the 50K Fana Axiom array [47] and compared mean AsA in the TT, CT and CC genotypic classes. Interestingly, the CC genotype, associated with higher AsA content in the Can×SS population (kk in Figure 4a), displayed about 13.3% significantly higher AsA content compared to the mean value of the CT and TT genotypes in the diverse accessions (Supplementary Fig. S5a), indicating the usefulness of this SNP for marker-assisted selection. Furthermore, a KASP assay was designed for this SNP, which resulted in almost identical genotypes, and again the CC genotype was associated with 12.2% higher AsA content (Supplementary Fig. S5b, c).

## Discussion

### Ascorbic acid content in strawberry fruit is environmentally and genetically controlled

As many fruit quality traits [45, 56, 57], fruit AsA content is strongly influenced by environmental conditions [42, 58]. Our results showed a clear environmental effect on AsA content but also a large phenotypic plasticity of Can×SS lines in the different countries and growing conditions. Due to the fact that AsA levels are affected by different abiotic factors, such as light and temperature, and by growing conditions and water availability [1, 6, 59], significant differences were observed in AsA content between countries (Table 1; Figure 2). Thus, Italy showed the lowest AsA levels in fruit (Table 1; Figure 2), which could be related to the clay-chalky soil (pH about 7) of the open field cultivation conditions at this location. Unlike other countries, the cultivation in France was in coconut fiber, in which there is a greater application of irrigation that may explain the lower dry matter content in the fruits. For this reason, AsA content in France increased when measured relative to DW (Table 1). These results suggest that the amount of water can significantly affect the concentration of AsA in the fruit. Accordingly, there seems to be a negative correlation between AsA content and fruit size [6] or weight [42].

Despite the environmental effect, an important genetic component was observed in the population (Supplementary Tables S2-S3). Other works have also shown a large variation in AsA content between *Fragaria* species and between cultivated strawberry varieties [35, 36, 60]. The heritability values in the Can×SS population were high in all five countries (Supplementary Table S3). High heritability values for this trait have been reported for other fruits such as kiwifruit and tomato [61, 62]. Differences in AsA content between parental and F_1_ lines (Table 1) and high heritability values (Supplementary Table S3) indicated that this population was useful for identifying QTLs controlling AsA content.

### QTL analysis for AsA content revealed a complex genetic architecture but a high number of stable QTLs

In addition to obtaining good collinearity of markers between the genetic map and the physical position in the reference *F. × ananassa* cv. ‘Camarosa’ v.1 genome (Supplementary Fig. S3) [30], we observed similar rearrangements reported in other works that used *F. vesca* or ‘Camarosa’ genomes [33, 47, 63], indicating a high quality of the Can×SS map. Indeed, a number of scaffolding errors in the ‘Camarosa’ genome, detected in our genetic map, were identified after comparison with the recently deposited ‘Royal Royce’ octoploid genome sequence [49].

A total of eight and seven QTLs for AsA content were detected in the Can×SS population depending on whether AsA concentration is expressed in fresh or dry weight (Figure 3). The mean variance explained by QTLs for AsA/FW and AsA/DW detected in our study was 12.8 and 13.8, respectively (Supplementary Table S5). Therefore, as previously reported for tomato [42, 64] and apple [14, 44], AsA content in strawberry fruit is affected by several loci with small effects. Although our results and other reports [42, 58] have shown that fruit AsA content is highly influenced by the environment, QTLs for ascorbic acid were fairly stable across environments, 50% for fruit AsA content measured in FW and 42.86% measured in DW. Stevens *et al*. [42], Mellidou *et al*. [14] and Zorrilla *et al*. [38, 45] also detected stable QTLs for this trait in tomato, apple and strawberry, respectively. Only one of the QTLs here identified in the Can×SS population, *qAsAFW-7C*, detected at two locations, was also detected in the 232 × 1392 population [38, 45]. Zorrilla-Fontanesi *et al*. [38] suggested two candidate genes in this genomic region based on the available *F. vesca* genome. However, none of them was found in our study in the corresponding region of the ‘Camarosa’ reference genome. High AsA content in fruits is a desired trait for consumers and positive alleles in markers here identified linked to stable QTLs could be successfully pyramided for early selection of progeny with increased levels.

### Candidate genes in AsA QTL intervals

The ultimate goal of a QTL analysis is to determine which genes are responsible for trait variation, which can be an arduous endeavor. One initial approach can be identifying candidate genes based on functional annotation and physical position. We focused our search on the 1-LOD intervals of the five stable QTLs and found a total of 4421 annotated transcripts. In this study, we have screened them based on their annotation and significant transcript expression in ripe fruit of ‘Camarosa’ which considerably reduced the list of candidate genes. With the exception of two *GGPs* that colocated with QTLs on LG 3A and 7C, no other genes with homology to any of the enzymes in the different AsA biosynthetic pathways were detected within the stable QTL regions. This result contradicted previous results in strawberry, tomato and apple where in addition to *GGP*, candidate genes such as *GME*, *GMP*, *GLDH*, *PMI* or *GalUR* localized within QTL intervals [14, 38, 42].

GGP catalyzes the first committed step and the main control point of the Smirnoff-Wheeler AsA biosynthetic pathway in *Arabidopsis* (Figure 1) [2, 10, 12]. While overexpression of other biosynthetic genes had rather limited success, *GGP* is the only gene that consistently increases AsA content in different species [12, 17, 18]. Of the two paralogs of *GGP* that colocated with AsA QTLs in our study, only *FaGGP(3A)* was significantly expressed in ripe fruit and considered a candidate gene associated with the QTL controlling AsA in FW and DW on LG 3A. Similarly, two paralogs of *GGP*, *MdGGP1* and *MdGGP3*, were associated with two QTLs for AsA content in apple [14]. In particular, differential expression of *MdGGP1* between low- and high-ascorbate cultivars further supported a key role of this gene in the natural variation observed for AsA content in apple. Our analysis of strawberry lines contrasting in AsA content revealed that the expression of *FaGGP(3A)* was more than two-fold higher in lines with high AsA content (Figure 6), supporting a role of this gene in regulating natural variation of AsA also in strawberry fruit.

Besides biosynthesis, the AsA pool is diminished by oxidation through the action of enzymes such as AO or APX, and increased by its recycling from DHA and MDHA by the Foyer–Halliwell-Asada cycle [25]. Two *MDHAR* and two *AO* colocated with stable QTLs for AsA content in our study. However, three of the candidate genes were not expressed in ripe ‘Camarosa’ fruits while *FaMDHAR(6B)* displayed low expression (Supplementary Fig. S4) and was not differentially expressed between F_1_ lines contrasting in AsA content (Figure 6) suggesting they might not be the underlying genes.

Candidate genes *FaGONST3(5A)* and *FaGONST3(5B)* on intervals for homoeologous QTLs for AsA content on LG 5A and 5B, have high similarity to the *Arabidopsis* primary Golgi GDP-*L*-galactose transporter *GGLT1*, which transports GDP-*L*-galactose from the cytosol to the Golgi, as a substrate for cell wall formation and thus competing with ascorbate biosynthesis [3, 27]. Accordingly, RNAi-mediated silencing of *GGLT1* in *Arabidopsis*, increased the availability of galactose in the cytosol as a substrate for ascorbate biosynthesis, leading to a raise of more than 50% in cytosolic AsA content [27]. As expected, based on the results on *Arabidopsis*, *FaGONST3(5A)* expression was significantly higher in lines with lower AsA/DW but no significant differences were observed in AsA relative to FW (Figure 6). In contrast, the expression of *FaGONST3(5B)* was higher in lines with higher AsA content, suggesting that this homoeolog might not be transporting GDP-*L*-galactose from the cytosol to the Golgi. Further molecular and functional analyses are needed to determine whether they encode functional Golgi-localized GDP-*L*-galactose transporters and their role on AsA concentration on strawberry fruit.

Candidate gene *FaPHT4;4(7C)* was identified on the QTL interval of *qAsAFW-7C*, detected at two locations, Germany and Italy. *Arabidopsis* AtPHT4;4 is a member of the phosphate transporter 4 family that is localized in the chloroplast membrane envelope and acts by transporting ascorbate from the cytosol to the chloroplast stroma [26, 65]. Knockout mutations on this gene results in reduced levels of AsA in leaves [26]. The strawberry *FaPHT4;4(7C)* homoeolog was predominantly expressed in green achenes and leaf tissue, although a constant level of expression was also detected during receptacle ripening (Supplementary Fig. S4). A two-fold increase in *FaPHT4;4(7C)* expression was detected by qRT-PCR in ripe fruits of strawberry lines with higher AsA content (Figure 6), suggesting that as in *Arabidopsis*, this gene might regulate AsA levels in strawberry.

### Allelic variation in *FaGGP(3A)* is associated with AsA content in strawberry fruit

KASP markers can efficiently detect SNP variations at a specific locus by PCR amplification and offer the advantage of analyzing many samples in a short time [66]. As modeled for the stable QTL *qAsAFW-3A*, four haplotypes of *FaGGP(3A)* were segregating in the Can×SS population and only the combination of b (GCT from ‘Candonga’) and d (ACT from ‘Senga Sengana’) haplotypes was associated with a 9.7% increase in AsA (Figure 7b). Taken together with the finding of a two-fold increase of *FaGGP(3A)* expression in lines with high AsA content (Figure 6), our results provide strong evidence that this gene regulates AsA content in strawberry. Furthermore, overexpression of kiwifruit *GGP* resulted in a two-fold increase in AsA content in strawberry fruit [17]. Our results agree with previous results in apple, where higher expression of specific *MdGGP1* allelic variants were also associated with higher total AsA concentration [14].

The KASP assays we developed for the three SNPs in *FaGGP(3A)* were not cross-predictive in the diverse germplasm collection, either because many haplotype combinations were present and limited the statistical power or because none of the three SNPs are the causal mutation. If *FaGGP(3A)* was the gene underlying the QTL on LG 3A, the causal SNP should be heterozygous in ‘Candonga’ and ‘Senga Sengana’, and therefore, none of the identified SNPs in the transcript, all segregating as a back-cross, could be the causal SNP. In many cases natural variation in traits is due to transcriptional changes in the causal gene [67], as occurs with the observed differential expression of *FaGGP(3A)* in lines with high or low AsA content, suggesting that the causal polymorphism, in linkage disequilibrium with the assessed SNPs, could be further away in the promoter region.

Nonetheless, the SNP AX-184662098 linked to the *qAsAFW-3A* QTL successfully predicted a 13.3% increase in AsA content in a diverse germplasm collection (Supplementary Fig. S5a). A KASP assay was developed for this SNP and shown to be a useful resource for strawberry breeders (Supplementary Fig. S5b, 5c), which will facilitate the selection of new strawberry cultivars with increased AsA content. Future studies and the development of predictive KASP or high-resolution melting (HRM) markers associated with the rest of the stable QTLs identified in this study are expected to further increase the predictive power which, as indicated by the pyramiding analysis, could represent a 30% increment in fruit AsA content.

## Materials and methods

### Plant materials

The Can×SS F_1_ population used in this study consisted of 126 lines generated from crossing cultivars ‘Candonga’ and ‘Senga Sengana’, adapted to contrasting Southern and Northern European areas, respectively. A subset of 35 F_1_ lines or the whole population was grown during 2016–2017 and 2017–2018 seasons at five locations across Europe: National Institute of Horticultural Research, (INHORT), Skierniewice, Poland (51°91’N 20°05′E); Hansabred GmbH & Co. KG, Dresden, Germany (51°09’N 13°47′E); INVENIO, Douville, France (45°02’N 0°61′E); Università Politecnica delle Marche, Ancona, Italy (43°32’N 13°22′E) and ‘El Cebollar’, Instituto Andaluz de Investigación y Formación Agraria y Pesquera (IFAPA), Huelva, Spain (37°14’N 06°48’W). Cultivation was carried out following conventional practices at each location. Ten plants from each genotype were grown in soil in all countries except in France, where strawberries were cultivated in coconut fiber in a soil-less tunnel. In Germany, Poland and Italy cultivation was in open field conditions while in Spain it was under macro tunnels. Analysis of segregation ratios of SNP markers (see Linkage mapping section) reduced the number of F_1_ lines to 113.

### Ascorbic acid quantification

AsA content was quantified in ripe fruits. At each location three biological replicates, of at least six fruits each, were harvested during the peak of the season, immediately frozen in liquid nitrogen and stored at −80°C. Samples were ground in liquid nitrogen and sent on dry ice to IFAPA Center at Málaga for AsA quantification. AsA content was determined by high-performance liquid chromatography (HPLC) as described previously [38, 68] using an HPLC system (Agilent series 1200, Agilent Technologies) equipped with a C18 column (Rx-C18, 4.6 x 100 mm, 3.5 μM, Agilent 861 967–902). The mobile phase consisted of 0.1 M NaH_2_PO_4_, 0.2 mM EDTA pH 3.1 [69] with a 0.7 ml/min flow rate and the detection was with a UV detector (G1315D, Agilent Technologies) at 254 nm. AsA content was calculated by interpolation from a standard curve plotted with known concentrations of AsA (Sigma) and expressed as mg AsA/100 g FW. Since there was a high variation in fruit size within the population and across the five environments, AsA concentration was also expressed as mg of AsA/g DW. For this, fruit dry matter content (DM; in percentage) of each line was calculated gravimetrically by drying fruit samples at 103°C for three days.

### Descriptive statistical analysis

Statistical analyses were run on *Statgraphics Centurion XVI* (StatPoint Technologies, USA) and *GraphPad Prism 8.0.0 for Windows®* (San Diego, California USA). Homoscedasticity and normality of trait’s distributions was evaluated by Levene and Kolmogorov–Smirnov, respectively. Data were analyzed by analysis of variance (ANOVA) and Student–Newman–Keuls for differences and comparison of means, respectively. Pearson correlation coefficients were calculated between different traits. Broad sense heritability [H^2^ = σ_G_^2^ / (σ_G_^2^ + σ_E_^2^)] was calculated in five countries for these traits from mean square values (MS) of ANOVA (MS*between* = σ_E_^2^ + nσ_G_^2^, MS*within* = σ_E_^2^; where σ_G_^2^ = genotypic variance, σ_E_^2^= environmental variance, and n = number of replicates = 3). H^2^ values were classified as high, medium and low using threshold >0.4, between 0.2 and 0.4, and < 0.2, respectively [70].

### Linkage mapping in the “Candonga” × “Senga Sengana” population

DNA was extracted from young leaves of F_1_ and parental lines using the CTAB method [71] with minor modifications. A total of 18 661 SNP markers were produced using the strawberry DArTseq platform [46] and 20 243 SNPs from categories Poly High Resolution (PHR) and No Minor Homozygous (NMH) were provided by the 50K Fana Axiom array [47]. SNPs were filtered for absence of missing values in parental lines, monomorphic markers in the progeny, markers that did not fit 1:1 or 1:2:1 segregations using the χ^2^ test (p = 0.05) and markers with average reproducibility or call rates below 0.975. Finally, markers with more than 5% missing scores were excluded, resulting in a total of 22 960 SNP markers, of which 8158, 10 464 and 4338 were maternal, paternal and heterozygous, respectively. In addition, six and two male and female markers were also included after genotyping the population with *ChFaM184*, *ChFaM061* [72] SSRs and marker *FaFAD1* for prediction of γ-decalactone in fruits [73, 74]. Markers were coded with the corresponding ‘Camarosa’ chromosome to aid LG identification and markers heterozygous in both parental lines were labeled with an initial “H”. The Can×SS linkage map was constructed using the *JoinMap^®^5* software [48]. To reduce the complexity of mapping, a single marker was selected among those markers that had identical genotypes in the population, preferably heterozygous and with the lowest number of missing data. The 7114 markers obtained were used for mapping using the Cross-Pollinated (CP) population coding type. Grouping was performed using independence Logarithm of Odds (LOD) and the default settings in *JoinMap^®^ 5,* and linkage groups were chosen at a LOD above eight for all but two (LOD 6 and 5) of the 30 groups obtained. The map was constructed using the maximum likelihood mapping algorithm, the Haldane function and the default parameters, except for increasing Chain length and stop criterion in S4, S5 and T0 to 4000, 8000 and 12 000. Isolated distorted markers were excluded from the map.

The seven HGs were named 1 to 7, as the corresponding LGs in the diploid *Fragaria vesca* v4 reference map [75] . DArTseq and Axiom SNP marker sequences were blasted to the published ‘Camarosa’ v1.0 genome [30] and assigned to the best-matching chromosome. LGs within homoeologous groups were then named according to the recent nomenclature of Hardigan et al. 2020 and 2021 using letters A, B, C and D in reference to species-derived subgenomes. Linkage maps were drawn using *MapChart 2.2* for Windows*^®^*.

### QTL analysis

QTLs for fruit AsA (estimated in mg/100 g FW and in mg/g DW) and %DM were detected using the integrated map of Can×SS population. To assess the stability of marker-trait associations, QTL analysis was carried out using *MapQTL^®^6* [76] and phenotypic data of each trait in each of the five countries separately, considering stable those QTL that were detected in at least two countries. To complement the analysis and based on high heritabilities, we also used the average AsA content and DM content across the five countries.

Data were first analyzed by the nonparametric Kruskal-Wallis (KW) test and markers with a level of significance higher than 0.005 were considered significantly associated with the trait. Second, Interval Mapping (IM) analysis was used to locate QTL positions in each LG using untransformed or transformed data, for traits deviating from normality. A permutation test was performed with 1000 permutations to estimate the significance LOD threshold for each trait. Regions with markers with LOD scores greater or equal than to the genome-wide limit value P ≤ 0.05 were considered QTLs. The most informative marker with the highest LOD score within each QTL was selected as co-factor to determine the location of QTLs more accurately by restricted Multiple QTL Method (rMQM) analysis. Markers were added and eliminated one by one as cofactors until they were stable. QTLs were named with a *q* followed by the trait abbreviation, and, in the case of QTLs detected in the countries separately, the country of origin and the name of the LG where it was detected. QTL position and confidence intervals were drawn using *MapChart 2.32* for *Windows^®^*.

### Candidate gene identification

We searched for candidate genes within the 1-LOD interval of stable QTLs using the *F.* × *ananassa* ‘Camarosa’ v1. 0 genome [30], available in the *Genome Database for Rosaceae* (https://www.rosaceae.org/). For stable QTLs detected on LG 3A (for AsA in FW and DW), LG 5A (for AsA in FW and DW) and LG 5B (for AsA in mg/g DW), candidate genes were searched within the 1-LOD interval defined by the QTL detected using the mean values across the five countries. For those LGs in which more than one QTL overlapped but no QTL was detected using the mean values, the 1-LOD interval of the QTL that covered more distance was taken. Genes with putative function related to AsA metabolism, transport or regulation were searched within those intervals. Then, and in order to reduce the number of candidate genes, their expression profile during fruit ripening was studied using data from a previous RNA-seq study [53] but mapped to the ‘Camarosa’ v1.0 a.2 genome [30]. Only genes with significant expression (>0.5 FPKM) in ripe fruit were selected for further studies.

### RNA isolation and gene expression analysis

Among the 113 F_1_ lines, we selected pools of fruits contrasting in mean ascorbate levels in the five countries. We extracted RNA from two pools of 10 F_1_ lines grown in Spain with high AsA content in FW (H037, H045, H078, H099, H102, H105, H107, H115, H119 and H123) and 10 with low AsA content in FW (H003, H006, H023, H036, H047, H054, H073, H079, H089 and H095), and other two pools with the highest (H010, H014, H037, H045, H052, H067, H076, H078, H107 and H110) or the lowest values (H006, H023, H036, H049, H079, H081, H089, H100, H103 and H125) of AsA in DW. Each pool consists of an equivalent amount of the fine powder previously ground (see *Ascorbic acid determination* part) of selected lines. Three biological replicates were produced and analyzed. Total RNA was isolated from strawberry fruit pools according to the method described by Gambino et al. [77] with minor modifications. A total of 900 μL extraction buffer (Tris–HCl 1 M, EDTA 0.5 M, CTAB 10%, PVP 10%, NaCl 5 M) supplemented with 18 μL β-mercaptoethanol was added to 300 mg fruit sample. After extraction and precipitation in isopropanol, RNA was resuspended in 30 μL of sterile H_2_O. Before reverse transcription, RNA was treated with DNAse Turbo (Invitrogen) according to the manufacturer’s instructions and chloroform precipitated. Retrotranscription was performed from 800 ng of RNA using the High-Capacity cDNA Reverse Transcription Kit (Applied biosystems by Thermo Fisher Scientific) in a S1000™ Thermal Cycler (Bio-Rad) following the manufacturer’s instructions.

Subgenome-specific primers were designed for transcript quantification of selected candidate genes by quantitative real-time PCR (qRT-PCR; Supplementary Table S7**)**. mRNA sequences of selected candidate genes and their *F. × ananassa* ‘Camarosa’ v1.0 [30] homoeologous copies were retrieved from the *Genome Database for Rosaceae* (https://www.rosaceae.org/). qPCRs were carried out in a CFX96™ Real-Time System (Bio-Rad) using 4 uL of a 1:10 dilution of the cDNA, 400 nM of each specific primer (Supplementary Table S7) and the SsoFast™ EvaGreen Supermix (Bio-Rad). Three technical replicates were used for each biological replicate. Reactions were performed for an initial denaturation at 95°C for 30 s, followed by 39 cycles of denaturalization at 95°C for 10 s and annealing at 60°C for 25 s. Relative expression of candidate genes was calculated by the 2^-ΔΔCT^ method using *FaGAPDH* and *FaDBP* as reference genes.

Statistical analysis was performed using GraphPad Prism version 8.0 software (San Diego, California, USA). The normality of trait’s distributions was evaluated by Kolmogorov–Smirnov. Data were analyzed by analysis of variance (ANOVA) and Tukey for differences and comparison of means, respectively.

### KASP marker development and analysis

To develop Kompetitive allele specific PCR (KASP) assays, SNPs were searched within the *FaGGP(3A)* transcribed region using RNAseq data obtained from a subset of lines from the Can×SS population. Two SNPs in the 5’UTR and one in the sixth exon were selected as targets based on high quality scores (Supplementary Table S8), higher coverage in all sequenced lines and compliance to the expected segregation ratio. Subgenome specific KASP primers (Supplementary Table S7) were designed using PolyOligo (https://github.com/MirkoLedda/polyoligo) with the ‘Camarosa’ v.1.0 reference genome [30]. KASP assays were conducted in 10 μL reactions containing 12.5 ng of template DNA, 5 μL 2× KASP-TF™ Master Mix (LGC Biosearch™ Technologies) and 0.14 μL primer assay mix (at 12 μM of allele 1-FAM, 12 μM allele 2-HEX and 30 μM reverse common primer). PCR was performed on a CFX96TM Real-Time thermal cycler (Bio-Rad) using the following conditions: 94°C for 15 min, 12 touchdown cycles of 94°C for 20 s and 63°C for 1 min decreasing 0.6°C per cycle, followed by 28 cycles of 94°C for 20 s and 57°C for 1 min and a final genotyping stage of 37°C for 1 min for plate reading. Two to four recycling programs consisting of three additional cycles were necessary for cluster improvement. Results were statistically analyzed by ANOVA and Tukey using the GraphPad Prism version 8.0 software (San Diego, California USA).

## Supplementary Material

Web_Material_uhad006

## Data Availability

All the data used in this study is provided in the Supplementary data.
