# Supplementary material for: QTL analysis for ascorbic acid content in strawberry fruit reveals a complex genetic architecture and association with GDP-L-galactose phosphorylase
Source: Hortic Res. 2023 Jan 19;10(3):uhad006. doi: 10.1093/hr/uhad006 (PMC10022485; doi:10.1093/hr/uhad006)
Supplement: Web_Material_uhad006 [file web_material_uhad006.zip › Suplementary Figures_R1.pdf]

**mg AsA/100g FW Germany**	0.54*	0.39*	0.64*	0.53*	0.68*	0.55*	0.28*	0.27*	0.49*	0.48*	0.02	0.16*	0.37*	0.11
	**mg AsA/100g FW Poland**	0.35*	0.43*	0.50*	0.47*	0.73*	0.29*	0.15*	0.40*	0.13*	0.42*	0.13*	0.27*	0.16*
		**mg AsA/100g FW France**	0.46*	0.37*	0.30*	0.31*	0.44*	0.24*	0.23*	0.15*	0.09	0.67*	0.23*	0.18*
			**mg AsA/100g FW Italy**	0.55*	0.53*	0.55*	0.41*	0.62*	0.38*	0.19*	-0.12*	0.14*	0.32*	0.21*
				**mg AsA/100g FW Spain**	0.38*	0.53*	0.28*	0.26*	0.47*	0.23*	-0.01	0.14*	0.25*	0.60*
					**mg AsA/g DW Germany**	0.54*	0.29*	0.34*	0.56*	-0.31*	-0.06	0.07	0.19*	-0.12*
						**mg AsA/g DW Poland**	0.33*	0.26*	0.54*	0.06	-0.32*	0.04	0.27*	0.06
							**mg AsA/g DW France**	0.34*	0.34*	0.02	-0.04	-0.35*	0.04	-0.04
								**mg AsA/g DW Italy**	0.19*	-0.04	-0.14*	-0.02	-0.52*	0.10
									**mg AsA/g DW Spain**	-0.04	-0.17*	-0.06	0.18*	-0.40*
										**%DM Germany**	0.09	0.14*	0.24*	0.29*
											**% DM Poland**	0.13*	0.02	0.15*
												**% DM France**	0.18*	0.21*
													**% DM Italy**	0.09
														**% DM Spain**

115 F_1 lines

Figure S1. Correlation matrix between AsA content in FW (mg AsA/ 100g FW) and in DW (mg AsA/g DW) and DM content (%DM) in the Can×SS population. *: indicates significant differences (p-value <0.05; Pearson Correlation).

(a)

	A	B	C	D	Total	Unique positions
**LG 1**	196	218	265	239	918	787
**LG 2**	344	314	372	88	1,118	970
**LG 3**	328	234	301	204	1,067	935
**LG 4**	131	164	171	321	787	677
**LG 5**	245	339	279	183	1,046	907
**LG 6**	373	336	234	241	1,184	1,026
**LG 7**	266	138	213	237	854	742
**Total**	1,883	1,743	1,835	1,513	**6,974**	**6,044**

(b)

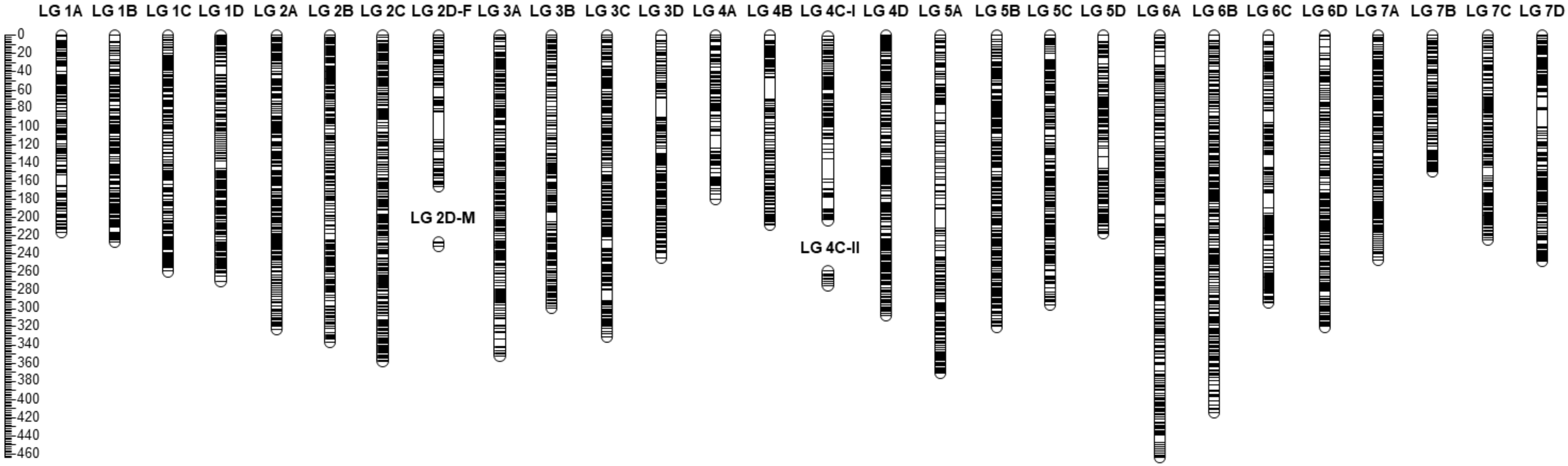

Figure S2. (a) Number of markers per chromosome in 'Candonga' x 'Senga Sengana' linkage map and (b) graphical representation of the 30 linkage groups (LG). LGs were named according to the recent nomenclature of Hardigan et al. 2020 and 2021 using letters A, B, C and D in reference to species-derived subgenomes: A, *F. vesca*; B, *F. iinumae*; C, *F. nipponica* and D, *F. viridis*.

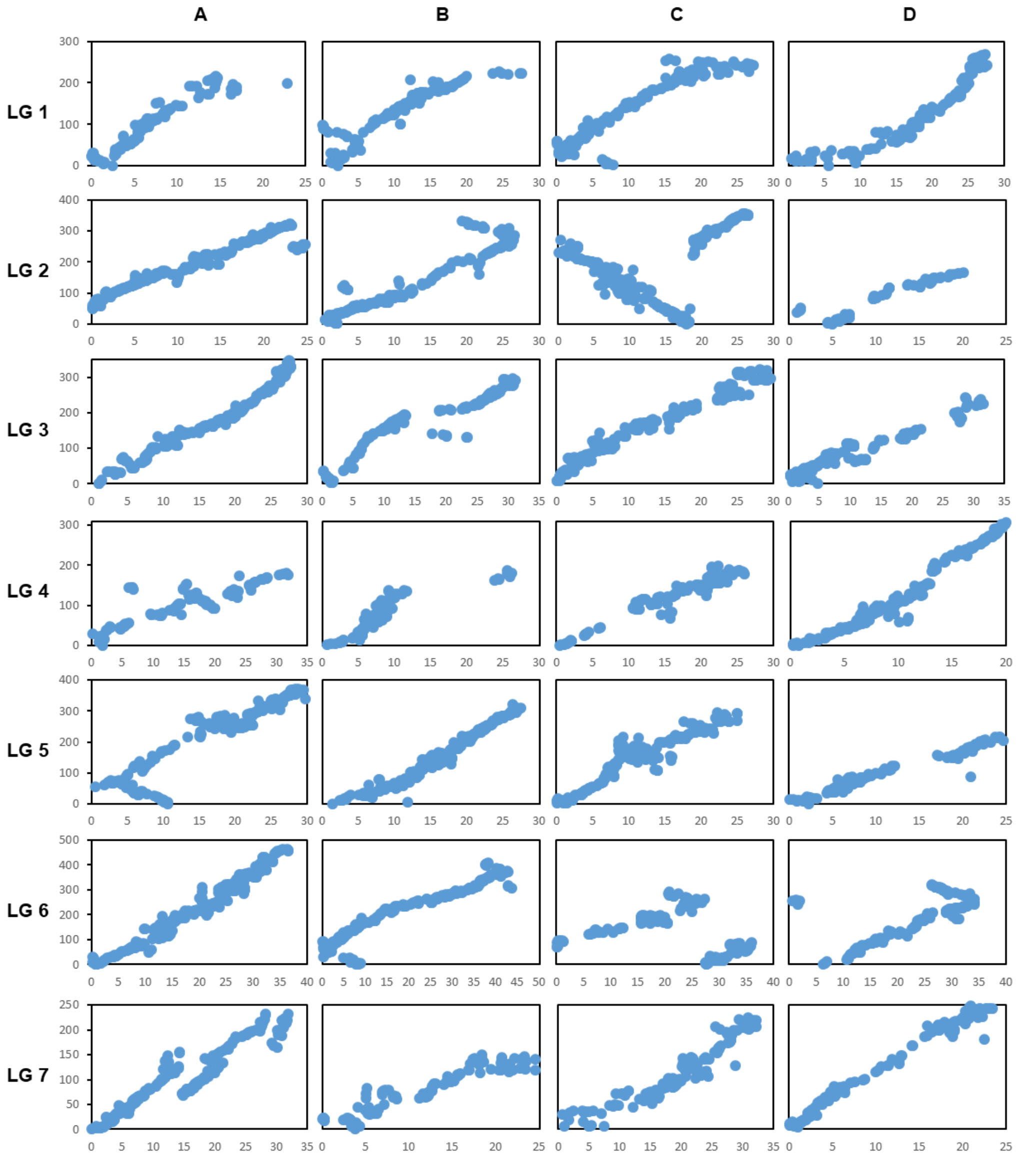

Figure S3. 'Candonga' x 'Senga Sengana' linkage maps aligned to 'Camarosa' genome sequence (Edger et al., 2019). Marker genetic distances (cM) on the y-axes plotted against 'Camarosa' physical positions (Mb) on the x-axes.

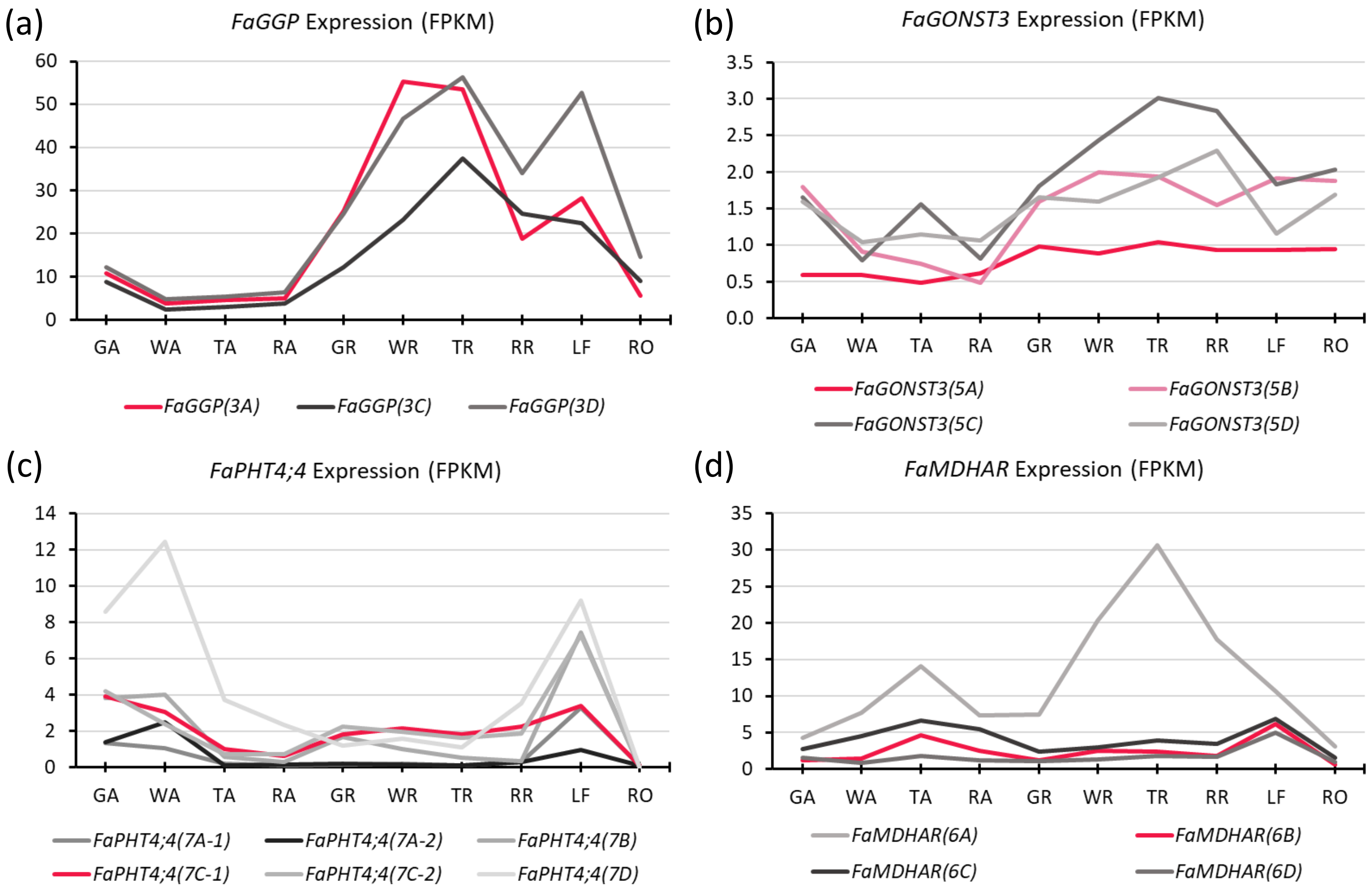

Figure S4. Expression (in FPKM) of candidate genes and their homoeologs from the different subgenomes. (a) *FaGGP* genes (GDP-L-galactose phosphorylase; *FaGGP(3A),* maker-Fvb3-4-augustus-gene-163.49; *FaGGP(3C),* maker-Fvb3-3-augustus-gene-128.36; *FaGGP(3D)*. (b) *FaGONST3* genes (GDP-mannose transporter GONST3; *FaGONST3(5A),* maker-Fvb5-1-augustus-gene-177.22; *FaGONST3(5B),* maker-Fvb5-3-augustus-gene-116.30; *FaGONST3(5C),* maker-Fvb5-4-snap-gene-146.50; *FaGONST3(5D),* maker-Fvb5-2-augustus-gene-166.43). (c) *FaMDHAR* genes (monodehydroascorbate reductase; *FaMDHAR(6A)*, maker-Fvb6-1-augustus-gene-23.39; *FaMDHAR(6B)*, maker-Fvb6-3-augustus-gene-20.23; *FaMDHAR(6C)*, snap_masked-Fvb6-2-processed-gene-289.21; *FaMDHAR(6D),* maker-Fvb6-4-augustus-gene-6.43). (d) *FaPHT4;4* genes (Ascorbate transporter; *FaPHT4;4(7A-1),* maker-Fvb7-2-augustus-gene-117.35; *FaPHT4;4(7A-2),* maker-Fvb7-2-augustus-gene-182.47; *FaPHT4;4(7B),* maker-Fvb7-3-augustus-gene-116.32; *FaPHT4;4(7C-1),* maker-Fvb7-1-snap-gene-175.49; *FaPHT4;4(7C-2),* maker-Fvb7-1-augustus-gene-188.48; *FaPHT4;4(7D),* snap_masked-Fvb7-4-processed-gene-111.36). Candidate genes detected in the QTL intervals are shown in pink and the rest of homoeologs are shown in grey. GA, Green achene; WA, White achene; TA, Turning Achene; RA, Red Achene; GR, Green Receptacle; WR, White Receptacle; TR, Turning Receptacle, RR, Red Receptacle; LF, Leaf; RO, Root.

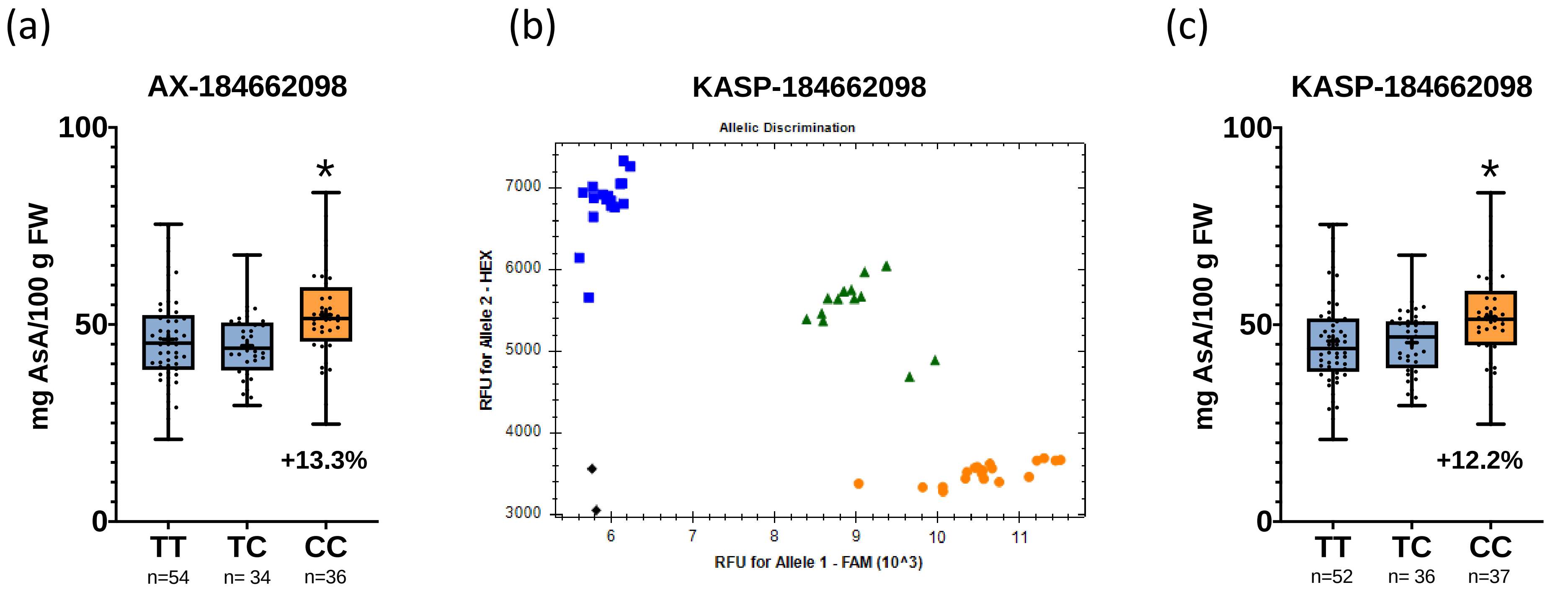

Figure S5. (a) Single marker analysis showing the effect on AsA content of positive (orange) and negative (blue) alleles for marker AX-184662098 in the diverse germplasm. (b) example of genotype clusters for KASP marker KASP-184662098. Genotype TT is labelled with FAM and CC with HEX. Heterozygous samples are represented in green and non-template controls in black. (c) Effects of KASP-184662098 genotypes on AsA content in the diverse germplasm. Boxes span the 25th and 75th percentiles and the middle line represents the median. Whiskers (T-bars) are the minimum and maximum values. Asterisk represent significant differences by ANOVA and multiple comparison (Tukey) analysis for the three possible genotypes (TT, TC, CC) of the selected marker (*: p-value < 0.05). *n*, number of lines.
